# Annotations

**Published:** 1899-02-04

**Authors:** 


### ANNOTATIONS.

### The Transmission of Typhoid Fever Poison Through the Air.

So much stress has been laid upon the fact that typhoid fever is in many cases a water-borne disease, and so definite has been the connection of great outbreaks—as, for example, that at Maidstone last year—with water supplies, that people are apt to forget how often the infection is carried in other ways. In *Public Health* Dr. John Brownlee records a series of cases of enteric fever which arose among patients in the City of Glasgow Fever Hospital, apparently from infection caught while within the hospital; and he traces the origin of the outbreak to dust carried to the patients' food from infected earth which had been thrown up in the repair of certain drains, which were known to have leaked into surrounding soil while containing enteric excreta. Dr. Brownlee gives another instructive instance of typhoid fever becoming endemic when implanted in a district the general sanitation of which was bad. How the fever is spread in such a district is pretty clear. The refuse from the ashpits is first emptied out into the street, and thence removed. The street is thus fouled, and a certain amount of this refuse being left behind is converted into dust, and scattered about by the wind. We have but to look at a basin of milk which has stood a single night to see how much dust is deposited from the atmosphere, and to understand that if such dust be specifically polluted there is no end to the vicious circle which keeps up not only the supply of the poison but its distribution. Thus by dust distribution we get a long continuing endemic prevalence of typhoid, very different from the short and sudden outbreak which is characteristic of the water-borne disease when it is spread by means of a public water supply.

### The Workmen's Compensation Act.

It may be as well to remind house surgeons that, according to the terms of the Workmen's Compensation Act, no obligation is laid upon the employer (or the insurance society representing him) to make use of the services of the house surgeon of any hospital for the purpose of examining or reporting upon any patient claiming compensation under the Act. It is provided in the Act that an injured workman "shall, if so required by the employer, submit himself for examination by a duly qualified practitioner, provided and paid by the employer," &c., but the employer may choose whomsoever he pleases. The problem then becomes a very complex one, when we remember that in many a case it would be quite impossible for the examining surgeon to ascertain for himself the precise condition of the parts without doing injury to the patient and retarding his recovery. The Act says that the injured workman must "submit himself for examination," but we doubt whether such submission could be made to imply consent to anything being done which would injure him; such, for example, as an examination of the condition of the fragments in a case of compound fracture, and if an examining surgeon attempted to do such a thing he would do it at his own peril. We think, then, that it will be far the best for all parties that it should become a generally recognised rule that the person to be appointed by the employer should be the surgeon who examined the injury on admission—that is, the house surgeon. Nothing could be worse than for employers to be encouraged to send in outsiders to examine patients. If they thoroughly examine them they do them harm, and if they do not examine them, but take the word of the house surgeon as to the condition of the parts, their certificates are worthless, and might at once be upset on cross-examination. For every reason then it is desirable that the house surgeon should in most cases be the "qualified practitioner provided and paid by the employer" to conduct the examination.

### The London Polyclinic.

With a roll of 400 annual subscribers the London Polyclinic or Medical Graduates College now occupies an assured position. How far complete success will be attained must no doubt depend upon the extent to which the physicians and surgeons attached to the large hospitals show themselves willing to co-operate in its work, for it is clear enough that a polyclinic carried on in entire independence of the hospitals would but be one more added to the numerous medical institutions among which the post-graduate has to grope his way. To those who know what is actually wanted by the post-graduate, and especially by the American and Colonial medical visitor to London, it is obvious that no arrangement which does not give them an actual *entrée* to the hospitals themselves is likely to be successful. So far it may be said that the existing schools have the whip-hand of the polyclinic. And that is true. But there is another side of the question. It is admitted on all hands that the crowds of English-speaking post-graduates who fill the benches of class after class in Berlin, Vienna, Marburg, and other German universities could be supplied in London with all they want if proper arrangements were but made for them It is probable indeed that there is material for a good post-graduate school in London even though its students had to be recruited from England alone. But those who visit foreign universities, and see how full the post-graduate classes are and note the sort of fees that are paid for instruction in the more advanced work which is taught in some of them, assure us that, as the phrase goes, "there is money in it," and we feel quite clear that if the managers of the new London Polyclinic are able, by a judicious choice of teachers, to make their classes really useful, and therefore popular, it will not be long before the staffs of the hospitals will see that it is greatly to their advantage to offer every facility to those post-graduates who may be introduced as polyclinic students. If once it can be arranged for the tutorial classes, the demonstrations, and the consultations to be carried out at the polyclinic itself, so that there shall be every day something of interest going on, and if at the same time those who enter there can feel that by so doing they will be enabled to visit the hospitals separately from the ordinary students, the institution ought soon to become one of the most attractive medical centres in Europe.

